# Supplementary material for: Validation of a MS Based Proteomics Method for Milk and Egg Quantification in Cookies at the Lowest VITAL Levels: An Alternative to the Use of Precautionary Labeling
Source: Foods. 2020 Oct 19;9(10):1489. doi: 10.3390/foods9101489 (PMC7603226; doi:10.3390/foods9101489)
Supplement: Supplementary file 1 [file foods-09-01489-s001.pdf]

Supporting Information

Table S1. Chromatographic conditions for milk and egg marker peptides separations.

**Column**	Perkin Elmer Aqueous C18 Column (2.1 x 150 mm; 3 μm; 100 Å)		
**Mobile phase**	Solvent A: H_2O + 0.1% Formic acid<br>Solvent B: Acetonitrile + 0.1% Formic acid		
	**Time (min)**	**%A**	**%B**
	0	90	10
	17	50	50
	17.2	10	90
	27	10	90
	27.2	90	10
	44	90	10
**Oven Temperature:** 30 ℃<br>**Flow:** 200 μL/mL<br>**Injection volume:** 10 μL			

Table S2. SRM conditions for the simultaneous detection of milk and egg marker peptides by QSight platform.

Protein	Peptide sequence	Precursor ion/charge	Retention time (min)	Acquisition Window (min)	Transition (m/z)	EV	CC L2	CE
αS1-casein	FFVAPFPEVFGK (FFV)	693.1 (+2)	13.1±0.02	12.59-13.59	920.3 (y_8^+)	15	-168	-26
					991.4 (y_9^+)	17	-168	-26
					1090.4 (y_{10}^+)	25	-172	26
	YLGYLEQLLR (YLG)	634.6 (+2)	12.5±0.01	11.98-12.98	658.2 (y_5^+)	31	-124	-28
					771.3 (y_6^+)	23	-152	-28
					991.4 (y_8^+)	24	-140	-27
β-Lactoglobulin	TPEVDDEALEK (TPE)	623.2 (+2)	5.8±0.01	5.31-6.31	572.5 (y_{10}^{2+})	35	-192	-29
					1047 (y_9^+)	45	-170	30
					819.1 (y_7^+)	41	-196	-29
	VLVLDTDYK (VLV)	533.2 (+2)	8.3±0.01	7.82-8.82	853 (y_7^+)	22	-100	-22
					640.8 (y_5^+)	24	-108	-24
					753.9 (y_6^+)	18	-100	-22
Vitellogenin-2	NIPFAEYPTYK (NIP)	671.6 (+2)	9.1±0.01	8.65-9.66	557.9 (y_9^{2+})	25	-140	-25
					507.9 (y_4^+)	33	-180	-39
					1114.9 (y_9^+)	25	-132	-23
	NIGELGVEK (NIG)	479.7 (+2)	6.5±0.01	6.01-7.01	673.9 (y_6^+)	27	-120	-21
					228.0 (b_2^+)	24	-88	-18
					544.8 (y_5^+)	27	-124	-24
Ovalbumin	ISQAVHAAHAEINEA GR (ISQ)	592.1 (+3)	4.3±0.01	3.80-4.80	778.5 (y_7^+)	42	-128	-31
					858.9 (y_8^+)	30	-204	-29
					545.9 (y_5^+)	20	-200	-40
	GGLEPINFQTAADQA R (GGL)	844.7 (+2)	8.9±0.01	8.44-9.45	1007.4 (y_9^+)	50	-272	-51
					1331.6 (y_{12}^+)	29	-336	-35
					1121.2 (y_{10}^+)	38	-380	-40

Table S3. Recovery table estimated with the aid of MoniQA reference materials incurred at low (3.54 $\mu g_{tot\ prot}/g_{matrix}$) and high (17.7 $\mu g_{tot\ prot}/g_{matrix}$) levels with milk ingredient.

Peptide/transition	LOW-MQA 102016 incurred material			HIGH-MQA 082016 incurred material		
	Measured concentration	*Validated concentration*	*Recovery*	*Measured concentration*	*Validated concentration*	*Recovery*
634.4→991.4	2,0±0,2	3,54	57±6 %	10,0±1,3	17,7	56±7 %
692.9→991.4	2,0±0,2	3,54	57±4 %	8,9±0,5	17,7	50±2 %

© 2020 by the authors. Licensee MDPI, Basel, Switzerland. This article is an open access article distributed under the terms and conditions of the Creative Commons Attribution (CC BY) license (http://creativecommons.org/licenses/by/4.0/).
